# When flames hit the brain, and the spark is far away: the role of PET-CT in diagnosing neurological Erdheim-Chester disease

**DOI:** 10.1055/s-0045-1802959

**Published:** 2025-02-28

**Authors:** Caio César Diniz Disserol, Guilherme Fleury Perini, Flávia Fernandes Silva Zacchi, Lívia Almeida Dutra

**Affiliations:** 1 Universidade Federal do Paraná, Hospital de Clínicas, Curitiba PR, Brazil.; 2 Instituto de Neurologia de Curitiba, Curitiba PR, Brazil.; 3 Hospital Israelita Albert Einstein, Instituto do Cérebro, São Paulo SP, Brazil.; 4 Grupo Fleury, Departamento de Anatomia Patológica, São Paulo SP, Brazil.

**Keywords:** Erdheim-Chester Disease, Histiocytosis, Positron-Emission Tomography-Computed Tomography, Proto-Oncogene Proteins B-Raf

## Abstract

Erdheim-Chester disease (ECD) is a rare histiocytic disorder that poses diagnostic and therapeutic challenges. Neurological manifestations are characterized by involvement of the meninges, brainstem, and/or cerebellum, and the differential diagnoses include sarcoidosis, IgG4 related disorders, autoimmune encephalitis, and high-risk syndromes. While present in a significant proportion of cases, neurological involvement is a predictor of mortality and may be the sole manifestation of the disease. In this paper, we discuss recent updates in histiocytic disorders and complementary diagnostic approaches, including positron-emission tomography-computed tomography (PET-CT), as guidance for biopsy in patients with neurological symptoms. Additionally, we explore how clinicians can interpret biopsy findings in conjunction with immunohistochemistry to guide targeted therapies, such as vemurafenib, for *BRAF* V600E mutation.

## CLINICAL VIGNETTE

A 77-year-old male patient with a history of heavy smoking presented with progressive dizziness and imbalance in the 15 days prior to consultation. His symptoms progressed after a couple of days to include dysarthria and diplopia, prompting hospital admission for further evaluation. There were no cognitive complaints; however, he reported an 8 kg weight loss in the 5 months prior to hospital admission.

Physical examination revealed an ataxic gait, ocular motor apraxia, normal extrinsic eye movements, normal muscle strength, brisk deep tendon reflexes, appendicular ataxia, and left hypoesthesia.

**Figure 1:**
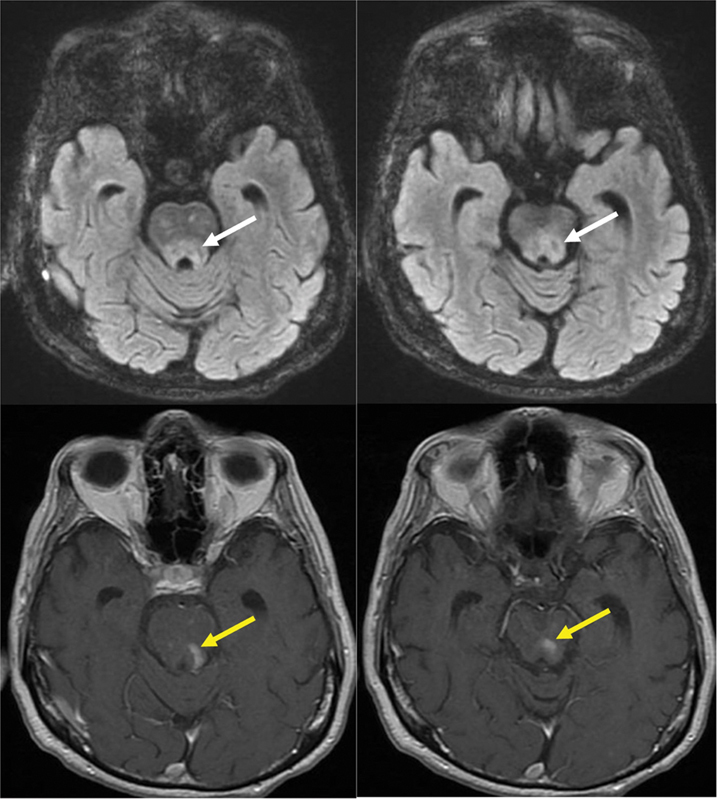
Brain magnetic resonance imaging showed a T2/fluid-attenuated inversion recovery brainstem lesion at the dorsal pontomesencephalic junction (white arrows) with contrast enhancement (yellow arrows).

Brain magnetic resonance imaging (MRI) revealed T2/fluid-attenuated inversion recovery (FLAIR) hyperintensities affecting the posterior brainstem and cerebellum, with contrast enhancement ( Figure 1 ). Perfusion MRI was normal, and proton spectroscopy revealed an increase in the choline/creatine ratio and a slight reduction in the N-acetyl-aspartate/creatine ratio. Extensive metabolic, hormonal, rheumatologic, and serologic panels were normal. Cerebrospinal fluid (CSF) analysis revealed < 1 cell/mm ^3^ , protein 36 mg/dL, negative oligoclonal bands (OCBs) and kappa-free light chain index. Cerebrospinal fluid immunophenotyping was normal, and metagenomic evaluation was negative. Moreover, high-risk antibodies were negative in serum and CSF.

**Figure 2:**
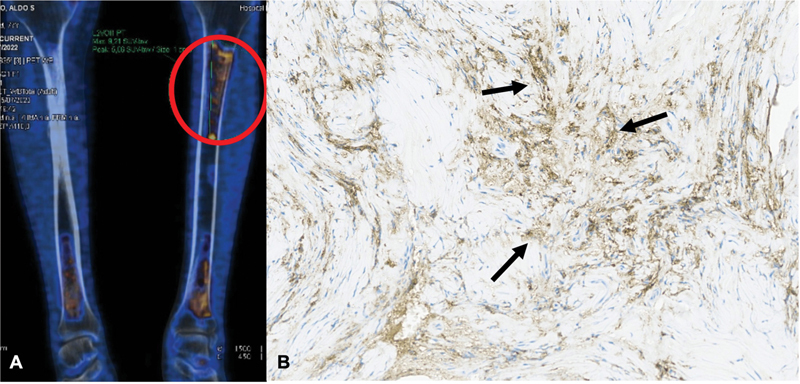
( **A** ) Positron-emission tomography-computed tomography showing an increased fluorodeoxyglucose-18F -uptake in the proximal left tibia (red circle). ( **B** ) Sheet of bone sample (30x) showing fibrosis and frequent cells with CD68 immunopositivity (black arrows); some cells appear elongated due to artifacts of cellular stretching.

At that time, the initial diagnosis was autoimmune encephalitis or a high-risk syndrome (rapidly-progressive cerebellar syndrome). The patient was treated with intravenous immunoglobulin and methylprednisolone, resulting in partial recovery. A PET-CT was subsequently performed, revealing increased fluorodeoxyglucose-18F (FDG) uptake in the pituitary gland and bilateral tibiae ( Figure 2A ). Although bone lesions were suggestive of infarcts, a bone biopsy was performed due to suspicion of histiocytosis.

Bone tissue samples showed intertrabecular spaces containing fibrous connective tissue surrounded by aggregates of histiocytes ( Figure 2B ). There was no evidence of epithelioid granulomas or necrosis. Immunohistochemistry was positive for CD68 and CD163 and negative for CD1a. Additionally, a *BRAF* V600E mutation was detected, confirming ECD.

The patient was treated with vemurafenib, a *BRAF* inhibitor commonly used in melanoma treatment. He presented full recovery and has remained asymptomatic.

## FROM PRESENTATION TO RESOLUTION: LESSONS LEARNED

### What are histiocytes and histiocytosis?

Histiocyte (derived from the Greek for “tissue cell”) is a general morphological term coined in 1913 to describe tissue-based macrophages. These cells are part of a group derived from myeloid progenitors known as the mononuclear phagocyte system (MPS), classically comprising monocytes, macrophages, and dendritic cells (DCs). 1 2 Although a reclassification of MPS based on cell ontogeny and function has been proposed, 3 histiocytosis remains an umbrella term to designate macrophage and DC disorders.

There are over 30 types of histiocytic disorders classified in 5 different groups based on cell origins, molecular pathogenesis, and clinical presentations. Langerhans-cell histiocytosis (LCH) and ECD are both DCs disorders classified within the L group, while Rosai-Dorfman disease (RDD) is a macrophage disorder categorized in the R group. These are the primary histiocytosis types affecting adults, though mixed presentations may occur. The main differences among these disorders are based on clinical grounds and pathological features ( Table 1 ). 4

**Table 1:** Clinical characteristics and differences among the primarily-adult histiocytic disorders

	ECD	LCH	RDD
**Organ involved**			
Bones	Long-bone (mainly around the knee) osteosclerosis at the metadiaphysis – **pathognomonic** (95%)	Osteolytic lesions, affecting mainly in the skull (60%)	Osteolytic lesions, affecting mainly the cortex (15%)
Nervous system	Mass-effect lesions in the brainstem and/or cerebellum. White matter lesions with gadolinium enhancement. Dural and pituitary stalk thickening (40%)	MRI T1 lesions in the globus pallidus/dentate nucleus or T2 lesions in the brainstem or cerebellum. Skull lesions involving the dura. Pituitary stalk thickening (5%)	Dural or parenchymal lesions (10%)
Endocrine	Any pituitary dysfunction (40–70%) DI, that may present years before diagnosis (40%)	Pituitary dysfunction (40–70%) DI, that may present years before diagnosis (20–30%)	Pituitary dysfunction rarely reported DI never reported
Respiratory	Mediastinal infiltration. Pleural, septal, and maxillary sinus thickening (50%)	Mostly seen in smokers. Pulmonary nodules or cysts (50–60%)	Involvement of large airways and sinuses. Interstitial pulmonary or sinus thickening. Pleural or pulmonary nodule (10–20%)
Dermatologic	Xanthelasma-like lesions around eyes, face, neck or inguinal folds (25%)	Xanthelasma-like lesions. Papular rash. Subcutaneous nodules (15–30%)	Subcutaneous nodules. Macular or papular rash (50%)
Cardiac	Right atrial and atrioventricular groove infiltration. MRI pericardial and myocardial infiltration (40–70%)	Rarely reported	Infiltration of the right atrium, interatrial septum, or left ventricle (< 5%)
Arterial	“Coated aorta” (periaortic infiltration). Infiltration of the supra-aortic trunk branches, visceral, renal, or coronary arteries (50–80%)	Rarely reported	Periaortic infiltration and carotid sheath (< 5%)
Retroperitoneum, including kidneys	“Hairy kidney” (perinephric infiltration); may have extension to renal pelvis and ureters causing renal failure. Adrenal infiltration (40–50%)	Rarely reported	Hilar masses. Subcapsular infiltration. Perinephric infiltration (5–10%)
Lymph nodes	Never reported	Isolated (5–10%)	Isolated or generalized lymphadenopathy (30–50%)
Orbits	Orbital masses (30%)	Never reported	Orbital masses (5%)
**Histopathologic features**			
CD68	+	+	−/+
CD163	+	−/+	+
S100	−/+	+/−	+
CD1a	−	+	−
*BRAF* V600E ^a^	+/−	+/−	−

Abbreviations: DI, diabetes insipidus; ECD, Erdheim-Chester disease; LCH, Langerhans cell histiocytosis; MRI, magnetic resonance imaging; RDD, Rosai-Dorfman disease.

Notes: ^a^ *BRAF* V600E testing by immunohistochemical analysis may have insufficient sensitivity to detect the mutant protein in histiocytic neoplasms. Molecular testing methods are recommended to definitely exclude a mutation.

Source: Adapted from: Goyal et al. ^4^

Historically, histiocytic disorders used to be considered inflammatory diseases. However, the identification of clonal cells and common mutations, such as *BRAF* V600E, has led to it being designated a neoplastic disease. 4 The V600E mutation of the *BRAF* gene was described in 2010 in LCH tissue lesions and was a game changer in the treatment of histiocytosis. BRAF is a kinase of the mitogen-activated protein kinase (MAPK) signal-transduction pathway, an important cascade composed by other kinases (RAS-RAF-MEK-ERK). 1 4

### What is ECD and the main systemic findings?

Erdheim-Chester disease is a very rare disorder, with ∼ 1,500 cases reported in the literature. It predominantly affects males (3:1) aged between 40 and 70 years. Pathologically, it is characterized by the presence of foamy histiocytes that are positive for CD68, CD163 and factor XIIIa, and negative for CD1a and Langerin (expression of S100 is variable). Approximately 50% of ECD patients harbor the BRAF V600E mutation, and nearly 40% exhibit other mutations in the MAPK pathway. 1 2 4

Erdheim-Chester disease is a multisystemic disease that can affect any organ ( Table 1 ). The most common initial symptom of ECD is bone pain in the inferior limbs. Osteosclerosis of the metadiaphyseal bones around the knees has been found in almost all patients and is pathognomonic. Positron-emission tomography-computed tomography is more sensitive than scintigraphy in detecting these lesions. 4 Other major systemic findings include fatigue, weight loss, fever, diabetes insipidus, and other hormone deficiencies (which may precede the diagnosis by decades), “hairy kidney” (retroperitoneal infiltration), “coated aorta” (periaortic infiltration), pleural or maxillary sinus thickening, right atrial pseudotumor, and normolipidemic eyelids xanthelasma. 1 4 5

Our patient exhibited the typical findings of ECD, including weight loss, fatigue, and involvement of the brainstem and cerebellum. Interestingly, pituitary involvement was documented only in PET-CT. Another important learning pearl from our case was the involvement of bilateral proximal tibiae found only in PET-CT, and very suggestive of bone infarction. Indeed, bone infarcts may occasionally show increased FDG uptake, 6 which may cause misinterpretation. For that reason, the presence of any type of bone lesions combined with neurological manifestations should raise the suspicion for ECD.

### When should ECD be suspected by neurologists?

Neurological involvement may be the only manifestation of the disease; it occurs in 20 to 50% of ECD patients and increases mortality even if asymptomatic. 2 7 8 It was first described in 1930 by Jakob Erdheim and William Chester in a 44-year-old male patient with diabetes insipidus, exophthalmos, and destructive bone lesions that pathologically presented lipid-rich granulomatous infiltration of various tissues with foamy histiocytes and multinucleated giant cells (Touton giant cells) within a fibrotic background, the reason they designated this condition as lipoid granulomatosis. 9

The main neurological symptoms of ECD include ataxia, cognitive impairment, headache, cranial nerve palsies, and peripheral neuropathy. Exophthalmos due to orbital masses may occur, and vascular infiltration may result in stroke. Clinical and MRI features of central nervous system (CNS) involvement in ECD may present three distinct patterns: tumoral infiltration (mainly with mass effect lesions) of the meninges, brain parenchyma/pituitary or spinal cord; pseudo-degenerative, with cerebellar or brainstem T2/FLAIR hyperintensities or cortical/cerebellar atrophy; and vascular, due to ischemic stroke or vascular sheathing. 5 7

Differential diagnoses of neurological ECD include sarcoidosis, granulomatosis with polyangiitis, IgG4 related disorders, fungal infections, and high-risk syndromes. In fact, the clinical presentation of our patient resembled a rapidly progressive cerebellar syndrome.

### How to investigate ECD and what is important for treatment?

**Figure 3:**
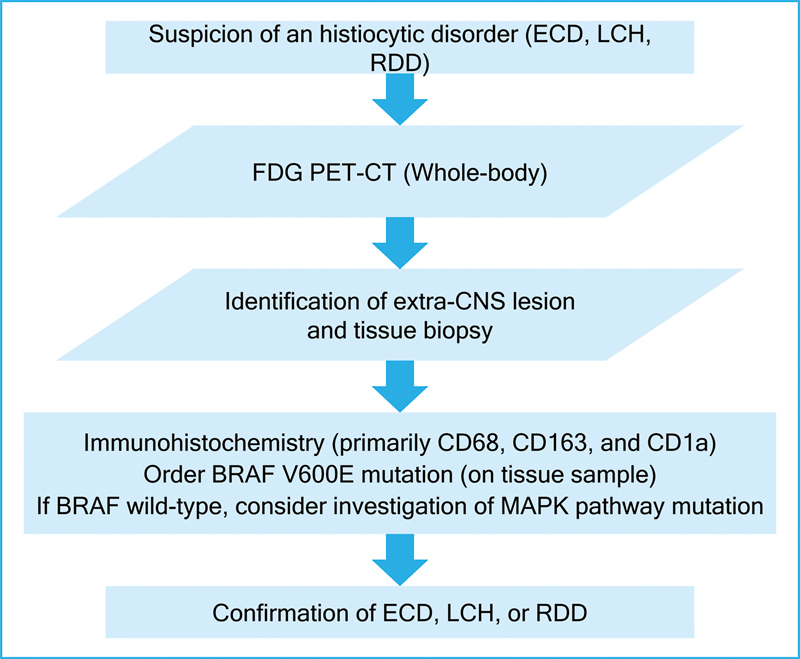
Suggested algorithm for the investigation of histiocytic disorders.

All histiocytic disorders are FDG-PET avid. Therefore, all patients with suspected or confirmed ECD should undergo PET-CT to identify the organs involved, locate biopsy sites and evaluate treatment response ( Figure 3 ). Bone scintigraphy is an alternative option but has lower sensitivity and does not access extra-osseous organs. 10

Biopsy should consistently include immunohistochemistry for CD68, CD1a, C163, and S100. Additional analysis for CD3, CD20, IgG4, and infectious agents is indicated to exclude differential diagnoses. If histocytes are identified, testing for the *BRAF* V600E mutation should be performed on tissue samples. If the mutation is absent, an expanded oncogene panel for additional MAPK pathway mutations is recommended, primarily due to the implications for treatment options. 1 4 5 11

Erdheim-Chester disease has carried a very poor prognosis, historically, with a 3-year fatality rate of 60%. Although *BRAF* V600E mutation is associated with cardiac and neurological involvement, 12 nowadays, these patients have excellent clinical response with vemurafenib. Additionally, cobimetinib, a MEK inhibitor, has proven highly effective for patients with *BRAF* wild-type ECD. 1

In conclusion, neurologists should show high suspicion for ECD in patients with cerebellar syndromes or unexplained neurological deficits with any type of bone lesions. Novel treatment options require additional knowledge of biopsy markers. Prompt diagnosis and treatment can halt the disease's progression and enable substantial clinical recovery, as demonstrated in our patient, who achieved complete remission with vemurafenib.

## Notes

TEACHING POINTS

1. Consider Erdheim-Chester disease (ECD) in patients presenting with neurological manifestations and bone lesions, even in cases suggestive of bone infarction.
2. Use positron-emission tomography-computed tomography (PET-CT) to investigate systemic involvement in patients with suspected neurological histiocytosis.
3. Request testing for *BRAF* V600E mutation in tissue biopsy samples to assess eligibility for targeted therapies, such as vemurafenib.
